# Aquamin, a marine derived multi-mineral, attenuates toll-like receptor-mediated inflammatory responses in macrophages

**DOI:** 10.3389/fimmu.2026.1901355

**Published:** 2026-08-04

**Authors:** Emmanouil Lioudakis, Sakshi Hans, Denise M. O’Gorman, Shane O’Connell, Margaret Lucitt

**Affiliations:** 1 Department of Pharmacology and Therapeutics, School of Medicine, Trinity College Dublin, Dublin, Ireland; 2 Marigot Research Centre, Sycamore Court, Tralee, Co., Kerry, Ireland

**Keywords:** Aquamin, innate immunity, macrophages, marine multi-mineral, toll-like receptors

## Abstract

Toll-like receptors (TLRs) play a central role in innate immune responses through recognition of pathogen-associated molecular patterns (PAMPs) and damage-associated molecular patterns (DAMPs). Activation of TLRs leads to the induction of inflammatory cytokines via myeloid differentiation primary response 88 (MyD88)- and TIR-domain-containing adaptor-inducing interferon-β (TRIF)-dependent signaling pathways. Aquamin, a multi-mineral supplement derived from the marine red algae *Lithothamnion* sp., is known for its anti-inflammatory properties. In this study, we demonstrate that Aquamin dose-dependently suppressed lipopolysaccharide (LPS) and Poly(I:C) production of pro-inflammatory cytokines [tumor necrosis factor-α (TNF-α) and interleukin (IL)-6] and chemokines in both human peripheral blood mononuclear cells (hPBMCs) and murine bone marrow-derived macrophages (mBMDMs), without causing cytotoxicity. The findings are consistent with Aquamin modulation of TRIF-associated signaling downstream of TLR3 and TLR4. These data provide a rationale for further investigations of Aquamin as a potential therapeutic intervention for inflammatory diseases associated with dysregulated TLR signaling.

## 1 Introduction

Toll-like receptors (TLRs) are evolutionarily conserved pattern recognition receptors (PRRs) that play a central role in host defense by recognizing pathogen-associated molecular patterns (PAMPs) and endogenous damage-associated molecular patterns (DAMPs) released during tissue injury (1–3). TLRs are expressed on the cell surface and within intracellular endosomal compartments of a wide range of immune cells, including macrophages, dendritic cells, and lymphocytes, as well as epithelial and stromal cells (2). Upon ligand recognition, TLRs initiate signaling cascades that coordinate innate and adaptive immune responses through the production of inflammatory cytokines, chemokines, and type I interferons (4).

Individual TLRs recognize distinct microbial ligands and activate specific downstream signaling pathways. TLR4 recognizes lipopolysaccharide (LPS), a major component of the outer membrane of Gram-negative bacteria, whereas TLR3 detects viral double-stranded RNA, which can be experimentally mimicked using the synthetic analogue polyinosinic-polycytidylic acid [Poly(I:C)]. TLR9 recognizes unmethylated CpG motifs commonly found in bacterial DNA (5–7). Following ligand binding, TLRs recruit adaptor proteins, principally myeloid differentiation primary response 88 (MyD88) and TIR-domain-containing adaptor-inducing interferon-β (TRIF), to initiate intracellular signaling (8). All TLRs, with the exception of TLR3, signal through MyD88, while TLR3 signals exclusively through TRIF. TLR4 is unique in activating both pathways, initially signaling through MyD88 at the plasma membrane before engaging TRIF following endosomal trafficking (9–12).

Activation of the MyD88 pathway results in rapid stimulation of nuclear factor-κB (NF-κB) and mitogen-activated protein kinases (MAPKs), promoting transcription of pro-inflammatory mediators including tumor necrosis factor-α (TNF-α), interleukin (IL)-6 and reactive oxygen species such as nitric oxide (NO) (10). In contrast, TRIF-dependent signaling activates interferon regulatory factor-3 (IRF3), resulting in type I interferon production while also contributing to sustained NF-κB and MAPK activation (11–13). Together, these signaling pathways coordinate antimicrobial defense while regulating immune cell recruitment and activation of adaptive immunity (14).

Although tightly regulated TLR activation is essential for protection against infection, excessive or prolonged signaling contributes to chronic inflammation and tissue injury. Activation of TLRs by endogenous DAMPs can drive sterile inflammation, a process implicated in autoimmune diseases, fibrosis, metabolic disorders, and cancer (15, 16). TLR4 signaling, in particular, has been implicated in persistent fibroblast activation and organ fibrosis and represents a potential therapeutic target in diseases such as systemic sclerosis (17). Within the gastrointestinal tract, TLRs are critical for maintaining epithelial barrier integrity and host–microbiota homeostasis; however, dysregulated TLR signaling has been linked to inflammatory bowel disease (IBD), impaired barrier function, and colitis-associated colorectal cancer (18–24). Recent studies have further highlighted the importance of regulating TLR-mediated innate immune signaling to control excessive inflammation while preserving protective immune responses, reinforcing TLR pathways as attractive therapeutic targets for chronic inflammatory diseases, including IBD (25–27).

Aquamin, is a multi-mineral supplement, derived from the red algae *Lithothamnion* sp. species, with evidence of anti-inflammatory effects (28, 29). It consists of an organo-mineral complex, consisting of 74 different minerals, and is especially rich in calcium and magnesium, with calcium and magnesium ratios of approximately 12:1. Aquamin is sold as a dietary supplement (GRAS 000028; Marigot Ltd., Cork, Ireland) used globally and has been reported to support significant improvements in intestinal health, by enhancing gut microbial diversity and barrier integrity (30). Significantly, Aquamin has also been found to suppress inflammation and to improve barrier function in gut organoid systems (31), and most recently Aquamin supplementation has been found to improve disease-related biomarkers in patients with ulcerative colitis (32). In addition to having positive impacts on intestinal health, Aquamin supplementation has also been shown to reduce liver ballooning degeneration and collagen deposition, while improving metabolic profiles, in mice on a high-fat diet (HFD) (33). While, collectively, these data provide evidence of Aquamin’s potential to modulate inflammation and provide protection in gut and liver disease states, the detailed cellular mechanistic pathways remain ill-defined.

To begin to address these questions, we investigated the immunomodulatory effects of Aquamin on innate immune signaling in primary murine macrophage cells and human peripheral blood mononuclear cells (hPBMCs). We demonstrate that Aquamin can significantly reduce both innate immune activation responses downstream of TLR4 and TLR3 across species. These effects occur in association with modulation of TRIF-dependent downstream signaling. Through dampening TLR4- and TLR3-driven pro-inflammatory signaling, Aquamin^®^ can modulate innate immune activation and limit excessive inflammatory mediator release. These data provide a potential biological basis for Aquamin’s reported protective effects in inflammatory disease studies.

## 2 Materials and methods

### 2.1 Aquamin^®^

Aquamin^®^ soluble is a mineral-rich natural product obtained from the calcified fronds of red marine algae of the *Lithothamnion* genus. It is particularly rich in calcium and magnesium (ratio approximately 12:1) and contains measurable levels of 72 additional trace elements (see **Supplementary Table 1** for the full mineral composition of Aquamin^®^) established via an independent laboratory (Advanced Laboratories; Salt Lake City, Utah). The complex mineral profile distinguishes Aquamin^®^ from single-mineral supplements. Aquamin is classified as Generally Recognized As Safe (GRAS) (GRN 000028). It is produced and supplied by Marigot Ltd., Cork, Ireland, and is marketed as a dietary supplement for use in a wide range of food and nutritional products. Aquamin^®^ has also received approval as an Investigational New Drug (IND No. 141600) from the U.S. Food and Drug Administration (FDA), permitting its clinical investigation and evaluation beyond its use as a dietary supplement. All materials used in the studies here, including biological agents, synthetic compounds, and the natural Aquamin product, were confirmed to be endotoxin-free. Microbiological testing results for Aquamin (Eurofins Ireland, Eurofins.ie), as documented in its certificate of analysis, are provided in **Supplementary Table 2**.

### 2.2 Isolation and culture of peripheral blood mononuclear cells

Buffy coats from anonymous donors were obtained from The Irish Blood Transfusion Service, National Blood Bank, St. James’s Hospital, James’s Street, Dublin 8. Peripheral blood mononuclear cells (PBMCs) were isolated and cultured, following institutional ethical approval. PBMCs were isolated from buffy coats as previously described (34) using density gradient Histopaque 10771 (Sigma-Aldrich, Poole, UK). Cells were resuspended and cultured in complete media [RPMI-1640 supplemented with 10% FBS, l-glutamine (2 mmol/L), penicillin (100 units/mL), and streptomycin (100 µg/mL)] all from Sigma-Aldrich (Poole, UK).

### 2.3 Isolation and culture of murine bone marrow-derived macrophages

Hind legs were dissected from adult C57BL/6 mice. The epiphyses of each femur and tibia were removed, so that the bone marrow could be accessed from the ends. Bone marrow from the tibiae and femurs were flushed into a 50-mL conical tube with 2–3 mL of complete media [DMEM supplemented with 10% FBS, 2 mM L-glutamine, 100 U/mL penicillin, and 100 μg/mL streptomycin (Sigma-Aldrich, Poole, UK)] using a 23-Gauge needle. The cell suspension was passed through a 70-μm pore size cell strainer. Bone marrow cells flushed from three individual mice were combined and centrifuged at 200 × *g* for 5 min. Cell pellets were resuspended in 10 mL of complete media supplemented with 20 ng/mL recombinant mouse macrophage colony-stimulating factor (rm M-CSF) (Miltenyi Biotec LTD), transferred to a T-75 flask, and incubated for 72 h in a humidified 5% CO_2_ cell culture incubator at 37°C. After 72 h, 5 mL of complete DMEM media supplemented with 20 ng/mL rm M-CSF was added and cells were incubated for a further 72 h before use.

### 2.4 Cell stimulation

Solutions of Aquamin (Marigot LTD) were prepared fresh prior to use in cell culture media depending on cell type. Aquamin was used at final concentrations ranging from 0.5 to 2 mg/mL, based on our previous *in vitro* studies demonstrating biological activity within this range without evidence of cytotoxicity. As calcium constitutes approximately 30% of Aquamin by weight, the highest concentration used (2 mg/mL) corresponds to an estimated calcium concentration of approximately 6 mg/dL, which is below the normal adult plasma total calcium range of 8.5–10.5 mg/dL (2.15–2.55 mmol/L). This physiological consideration also informed the selection of the upper concentration for the *in vitro* experiments. The following TLR agonists were used: TLR3 Poly(I:C) HMW (100 μg/mL), TLR 9 CpG ODN 1826 (3 μM) (both InvivoGen), and TLR4 LPS (100 ng/mL) from *Escherichia coli* O55:B5 (Sigma-Aldrich, L6529). hPBMCs or murine bone marrow-derived macrophages (mBMDMs) were first treated with Aquamin (0.5, 1, and 2 mg/mL) for 3 h prior to TLR agonist stimulation for a further 4–12 h. Following TLR stimulation, cell culture media was harvested and stored at −80°C for enzyme-linked immunosorbent assay (ELISA) cytokine measurements. Cells were processed for either protein or RNA extraction or fixed for immunofluorescence staining as described below.

### 2.5 Cell viability

The Alamar blue assay was used to access cell viability using Resazurin Sodium Salt (Invitrogen R12204). Cells were plated at 100,000/well in a 96-well plate and treated with various concentrations of Aquamin S (0.5–2 mg/mL dissolved in culture media) and incubated for 12 h. At the end of the incubation period, resazurin solution equal to 10% of the cell culture volume was added to each well and incubation continued in standard culture conditions for a further 4 h at 37°C. The absorbance at 570 nm was read on a BioTek ELx808 using KC Junior software.

### 2.6 Semi-quantitative RT-PCR

RNA was extracted from cells using the Bioline RNA extraction kit (London, UK) following standard kit protocols. RNA concentration and purity were determined using NanoDrop 2000 (Life Technologies Ltd., Paisley, UK). RNA samples underwent a DNase 1 (Invitrogen) treatment step, to digest single- and double-stranded DNA. The mRNA sample was then ready to use in reverse transcription reactions for cDNA synthesis using the RevertAid Reverse Transcriptase (Thermo Fisher Scientific, EP0442) kit. cDNA was used for targeted gene expression analyzed by semi-quantitative RT-PCR using target-specific primers. See **Supplementary Table 3** for all primer sequences (Integrated DNA Technologies/Belgium). RT-PCR was carried out using SYBR green GoTaq DNA polymerase (Promega, A6002) on the Mx3000P QPCR System (Agilent Technology), 40 cycles at 95°C for 15 s, 62°C for 1 min, and 72°C for 15 s with a final hold step at 4°C. The size of the amplicons was checked using a dissociation curve added to the end of the PCR run for primer sequence specificity. Gene expression fold changes was calculated using the comparative Ct method (2^−ΔΔCt^) and normalized to a reference housekeeping gene GAPDH or 18S RNA. Housekeeping genes were selected based on their stable expression under the respective experimental conditions here. No significant changes in the expression of either reference gene were observed following Aquamin treatment under the conditions examined.

### 2.7 SDS-PAGE and Western immunoblotting

Cell lysis and protein extraction were carried out using RIPA buffer (Trizma Base 50 mM; NaCl 150 mM; EDTA 2 mM, NP-40 0.5% S) supplemented with 1× protease/phosphatase inhibitor cocktail (PPC2020 Signa Aldrich) on the day of use. The Pierce BCA (Thermo Fisher Scientific) kit was used to quantity total protein according to the manufacturer’s instructions. Standard Western blot techniques were performed as previously described (34). Briefly 20 μg of total protein was resolved on 12% Tris-glycine SDS-PAGE gels along with a molecular weight standard [Fisher BioReagents (BP3603-500), EZ-Run™ Pre-stained Protein Ladder, 10–170 kDa]. The protein resolved gels were transferred using semi-dry gel transfer onto a PVDF membrane (Amersham, Buchinghamshire, United Kingdom). Following the transfer, the PVDF membrane was blocked for 2 h with TBS-T containing 5% w/v Marvel and probed overnight at 4 °C with the following primary antibodies: Primary anti mouse rabbit mAb antibodies all at 1:1,000 dilution from Cell Signaling Technology: iNOS (13120S), IκBα (4812), phospho-NF-κB p65 (Ser536) (3033), and NF-κB p65 (8242). This was followed by secondary antibody detection using Anti-rabbit IgG, HRP-linked Antibody (1:5,000) (7074), Cell Signaling Technology. Mouse beta-Actin Antibody (C4) (sc-47778) HRP conjugated (Santa Cruz Biotechnology, Santa Cruz, CA, USA)] at 1:1,000 dilution was used as a loading control. Signal detection was performed via enhanced chemiluminescence Pierce™ ECL (Thermo Fisher Scientific) and autoradiography using a Fusion Fx imaging system (Vilber). Densitometry quantification of immune-detected bands was carried out using Bio1D software.

### 2.8 Enzyme-linked immunosorbent assay

The concentration of cytokines present in cell culture supernatants was quantified using the following ELISA kits according to manufacturer’s instructions: Human TNF-α (DY210) and IL-6 (DY206) from R & D Systems (Minneapolis, MN, USA) and Mouse TNF-α (88-7324-88) and IL-6 (88-7064-88) from Invitrogen. Samples were measured in triplicate, and absorbances were read on a BioTek ELx808 using KC Junior software.

### 2.9 Cell immunofluorescence staining

Cells were fixed in 4% paraformaldehyde (PFA) for 20 min at room temperature and blocked with 2% BSA and 0.3% Triton X-100 for 1 h. The cells were incubated at room temperature for 90 min with primary antibodies and followed by secondary antibody for 60 min, all at room temperature and counterstained with DAPI (4083) nuclear staining. The primary antibodies include iNOS Rabbit mAb (1:200, 13120S) and IRF-3 Rabbit mAb (1:200, 4302) with secondary antibody detection using Anti-rabbit IgG (Alexa Fluor^®^ 555 Conjugate) (4413), all from Cell Signaling Technology. Cell images were acquired using an inverted light microscope (Zeiss Axiovert 200, Carl Zeiss) and immunofluorescence was quantified using ImageJ software.

### 2.10 Statistical analysis

Results were graphed and statistical analysis was performed using GraphPad Prism Version 9 (GraphPad Software Inc., California, USA). All experiments represent independent biological replicates performed on separate cell preparations, with technical replicates averaged prior to statistical analysis. Data are presented as the mean ± standard error of the mean (SEM) of independent biological replicates unless otherwise stated. Statistical comparisons between two groups were performed using an unpaired Student’s *t*-test, while comparisons among three or more groups were analyzed by one-way analysis of variance (ANOVA) followed by Dunnett’s *post-hoc* test for multiple comparisons. A *p*-value of <0.05 was considered statistically significant and is indicated by an asterisk in the figures.

## 3 Results

### 3.1 Aquamin inhibits LPS-driven TLR4-mediated pro-inflammatory cytokines in both primary human PBMCs and murine BMDMs

**Figure 1:**
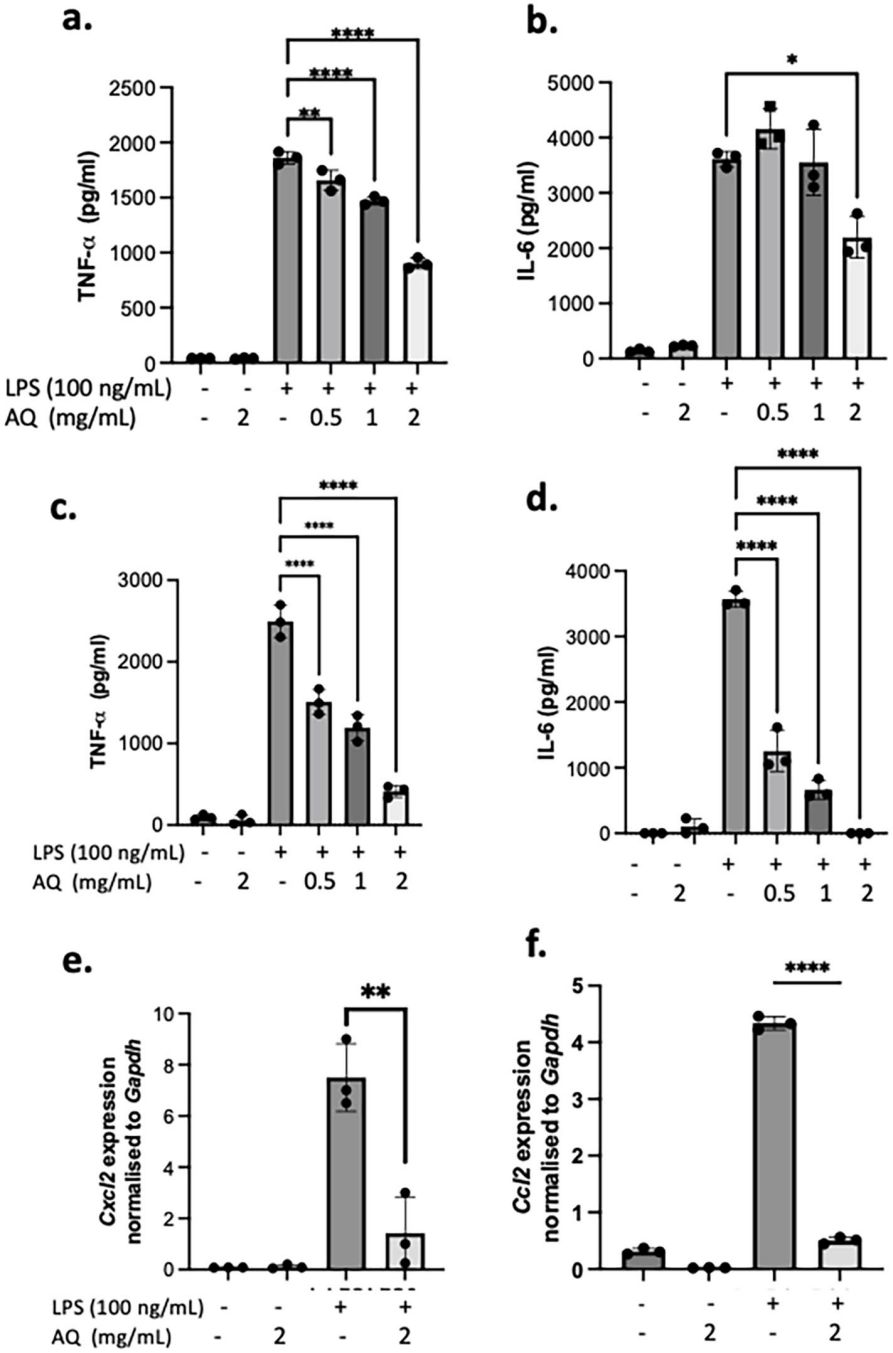
Aquamin inhibits inflammatory cytokines and chemokines downstream of LPS-driven TLR 4 signaling in PBMCs and murine BMDMs. Human PBMCs were pretreated with Aquamin (0.5, 1, and 2 mg/mL) for 3 h followed by stimulation with LPS (100 ng/mL) for 12 h. PBMC cell culture cytokine levels of **(a)** TNF-α and **(b)** IL-6 detected by ELISA. Murine BMDMs were pretreated with Aquamin (0.5, 1, and 2 mg/mL) for 3 h and exposed to LPS (100 ng/mL) for 6 h followed by total cellular RNA extraction and 12 h followed by collection of cell culture supernatants. BMDM cell culture cytokine levels of **(c)** TNF-α and **(d)** IL-6 detected by ELISA. BMDM mRNA expression levels of **(e)** *Cxcl2* and **(f)** *Ccl2*, normalized to *Gapdh* and expressed relative to untreated cells. Data presented as mean ± SEM from three independent experiments *p < 0.05,**p < 0.01, ****p < 0.0001; vs. LPS alone using one-way ANOVA statistical test.

**Figure 2:**
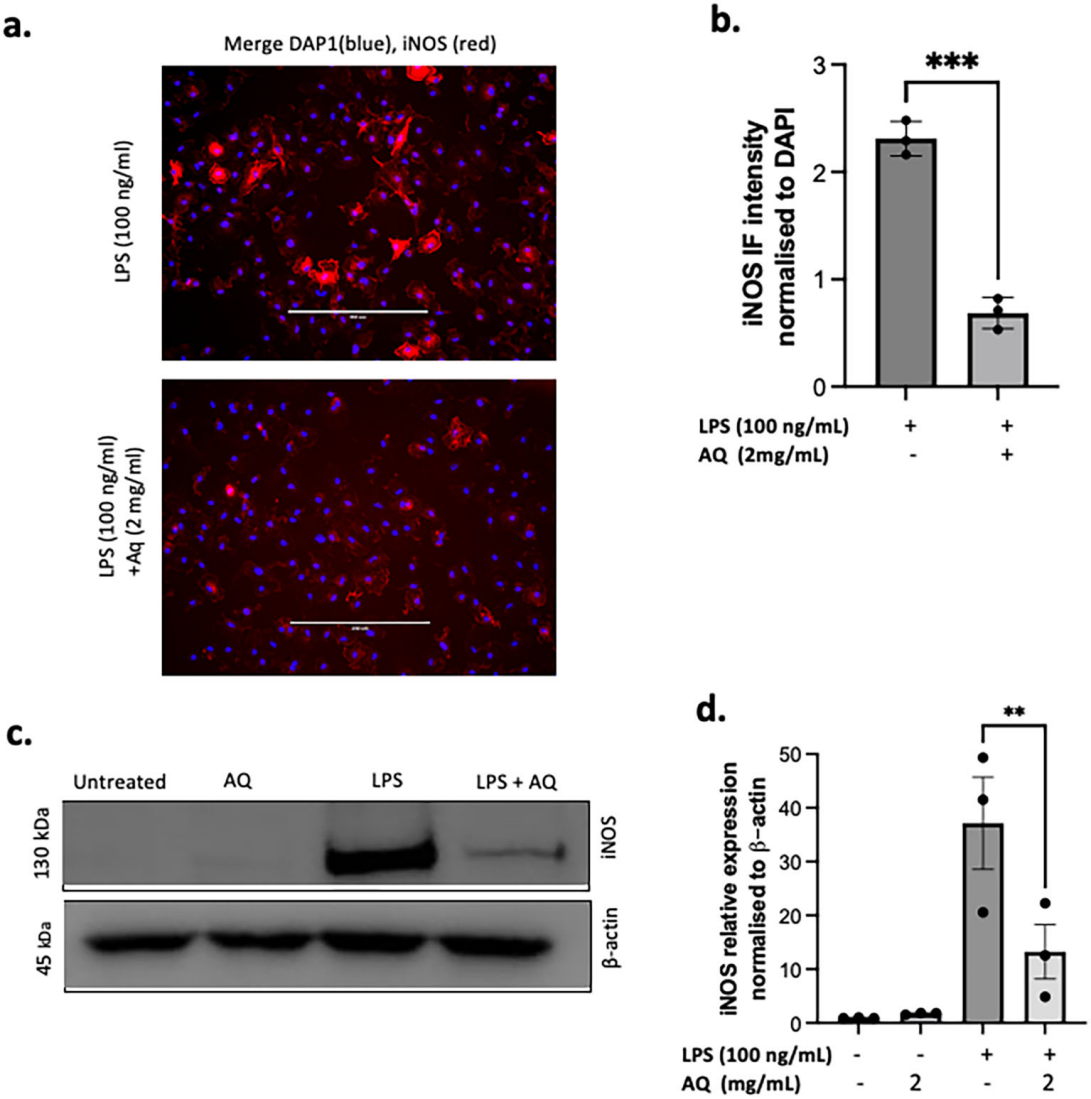
Aquamin inhibits LPS-induced iNOS expression in murine BMDM. BMDMs pretreated with Aquamin (2 mg/mL) for 3 h followed by stimulation with LPS (100 ng/mL) for 12 h. **(a)** Representative immunofluorescence (IF) overlay of iNOS protein abundance (red) and DAPI nuclear stain (blue) in BMDMs; scale bar, 200 μm. **(b)** Bar graph of iNOS IF intensity normalized to DAPI. **(c)** Representative Western blot protein expression of iNOS from BMDM total protein lysates. **(d)** Bar graph of densitometry analysis for iNOS protein expression levels normalized to β-actin presented as fold change in protein expression relative to untreated control cells. Data presented as mean ± SEM, *n* = 3, **p < 0.01, ***p < 0.001 versus LPS alone using one-way ANOVA statistical test.

To investigate whether Aquamin alters innate immune activation, we first assessed its ability to modulate pro-inflammatory cytokine production in hPBMCs, a heterogeneous population of immune cells, and in primary mBMDMs. Initially, we examined whether Aquamin affects the release of cytokines associated with LPS TLR4 signaling in primary hPBMCs. The effects of Aquamin treatment, followed by LPS stimulation, on cytokine levels in hPBMCs were measured in cell culture supernatants using ELISAs. Upon LPS stimulation, Aquamin dose-dependently reduced the secretion of TNF-α (**Figure 1a**) and IL-6 (**Figure 1b**) after 12 h. Similarly, an Aquamin dose-dependent inhibitory effect was also observed in mBMDMs, with TNF-α (**Figure 1c**) and IL-6 (**Figure 1d**) reduced at 12 h post LPS stimulation. This effect was also evident at the protein [Supplementary TNF-α (**Figure 2b**) and IL-6 (**Figure 2d**)] and gene expression level [Supplementary *Tnfa* (**Figure 2a**) and *Il6* (**Figure 2b**)] 6 h post LPS stimulation.

C-X-C Motif Chemokine Ligand 2 (CXCL2), also referred to as MIP-2α, and C-C Motif Chemokine Ligand 2 (CCL2), also known as MCP-1, are chemokines that attract and orchestrate immune cells including neutrophils [CXCL2 (35)] and monocytes/macrophages [CCL2 (36)] to sites of injury or infection. Consistent with its inhibitory effects on cytokine secretion, Aquamin pretreatment of mBMDMs also reduced the expression of both *Cxcl2* (**Figure 1e**) and *Ccl2* (**Figure 1f**) following LPS stimulation. In all cases, Aquamin treatment alone did not alter the induction of TNF-α (**Figures 1a, c**), IL-6 (**Figures 1b, d**), or *Cxcl2* (**Figure 1e**) and *Ccl2* (**Figure 1f**) expression. Importantly, these inhibitory effects of Aquamin were not as a result of cellular toxicity, as both PBMCs and BMDMs treated with varying doses of Aquamin (0.5, 1, and 2 mg/mL) for 12 h showed no reduced cell viability compared to untreated cells (**Supplementary Figures 1a, b**). These results clearly demonstrate that Aquamin can reduce LPS/TLR4-driven innate immune activation of both hPBMCs and primary murine macrophages without compromising cell viability.

### 3.2 Aquamin inhibits LPS-induced iNOS in murine BMDM

To further explore the effects of Aquamin on innate immune activation, we next examined its potential to alter inducible nitric oxide synthase (iNOS) expression. iNOS is an enzyme that catalyzes the production of NO in mammalian cells. NO is an important mediator of cellular inflammation involved in pathogen killing, and its activity is known to be regulated by several proinflammatory cytokines, as well as PAMPs such as LPS (37). The impact of Aquamin treatment on production of iNOS was investigated here using antibody immunofluorescence (IF) detection of iNOS in LPS-stimulated BMDMs. Notably, treatment of BMDMs with LPS in the presence of Aquamin (2 mg/mL) resulted in a decreased IF intensity of iNOS staining compared to LPS stimulation alone (**Figures 2a, b**). Consistent with IF staining, iNOS protein expression levels from whole cellular lysates was quantified by Western blot (**Figures 2c, d**), demonstrating that the protein expression levels of iNOS were significantly reduced in Aquamin LPS-treated BMDMs compared to LPS treatment alone. Importantly, Aquamin treatment alone was not found to alter iNOS protein expression in comparison to untreated cells. Interestingly, iNOS is a hallmark of pro-inflammatory macrophages, which produce large amounts of NO and other reactive nitrogen species (RNS) (38). These data indicate that Aquamin can reduce iNOS expression, which is consistent with attenuation of pro-inflammatory activation.

### 3.3 Aquamin inhibits TRIF gene expression downstream of TLR4 stimulation in murine BMDMs without affecting IκBα degradation and phosphorylation of p65 in murine BMDM

**Figure 3:**
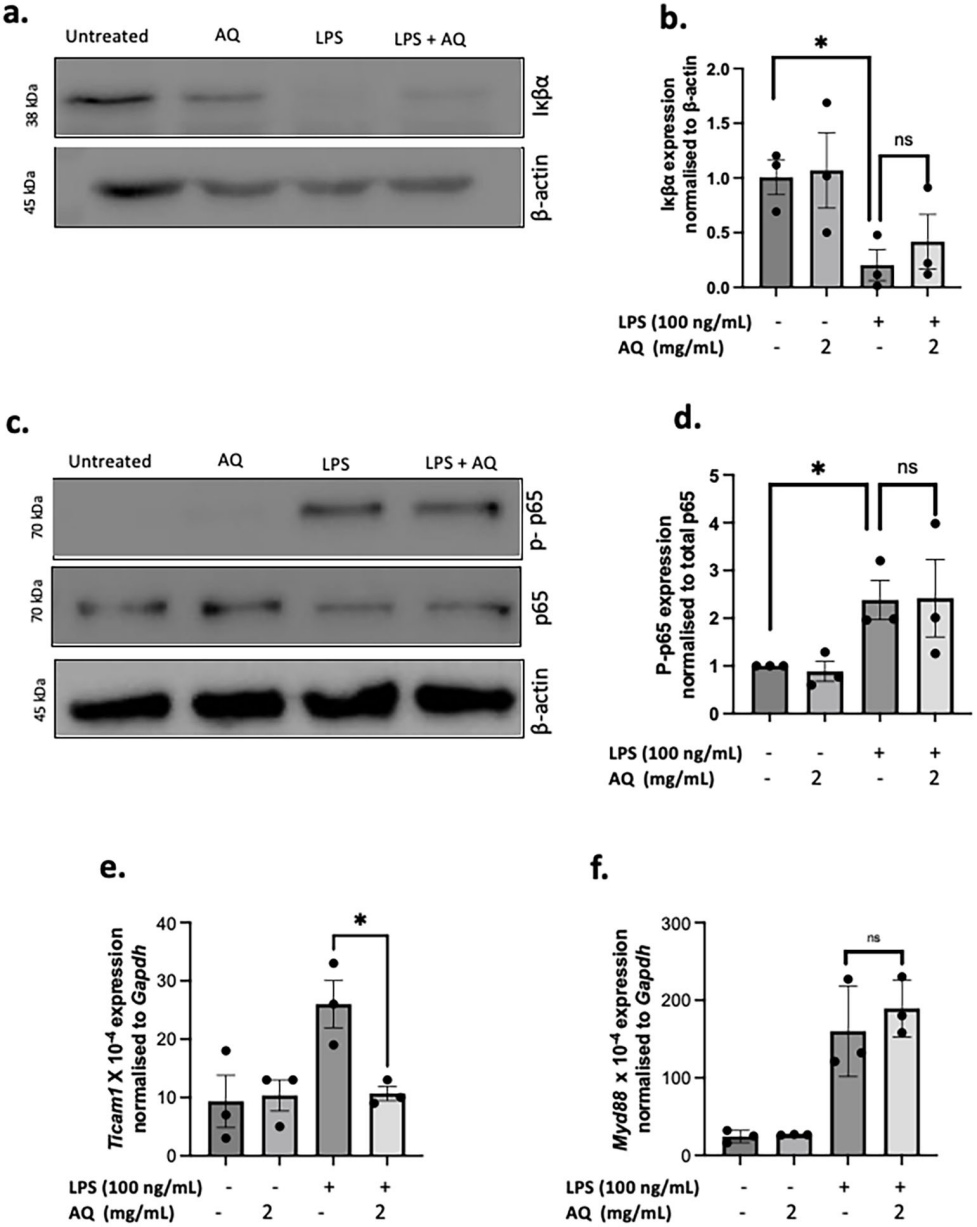
Aquamin inhibits LPS-induced *Ticam1* without affecting *Myd88* gene expression, IκBα degradation, and phosphorylation of p65 in murine BMDM. BMDMs pretreated with Aquamin (2 mg/mL) for 3 h followed by stimulation with LPS (100 ng/mL) for 15 min to detect expression of IκBα and 7 min to detect expression of phosphorylated (p)-p65 in total cell protein lysates. Representative Western blot protein expression of **(a)** IκBα 15 min post LPS stimulation and **(c)** p-p65, total p65, and β-actin 7 min post LPS stimulation. Bar graph of densitometry analysis for **(b)** IκBα and **(d)** p-p65 protein expression levels normalized to β-actin and total p65 protein loading and presented as fold change in protein expression relative to untreated control cells. BMDMs pretreated with Aquamin (2 mg/mL) for 3 h and exposed to LPS (100 ng/mL) for 4 h followed by total RNA extraction. **(e)** *Ticam1* and **(f)** *Myd88* gene expression normalized to *Gapdh* and expressed relative to untreated cells. Data presented as mean ± SEM, *n* = 3, *p < 0.05 vs. LPS alone using one-way ANOVA statistical test.

Similar to TNF-α and IL-6 cytokine production, LPS induction of TLR4-driven iNOS production occurs through activation of the NF-κB transcription factor. It is well established that NF-κB activation plays a crucial role in the signaling mechanisms downstream of LPS/TLR4 in macrophage cells, driving inflammatory cytokine release (7). This activation occurs via NF-κB p-65 subunit phosphorylation, allowing its translocation to the nucleus to initiate transcription of regulated genes. In unstimulated cells, IκBα sequesters NF-κB in the cytoplasm, keeping NF-κB inactive. Upon LPS/TLR4 signaling, phosphorylation of IκBα occurs, allowing dissociation from the NF-κB complex and IκBα targeting for proteasomal degradation (39). To further investigate the anti-inflammatory effects of Aquamin described above, we assessed how Aquamin might impact early-stage LPS-induced NF-κB transcriptional activation. Interestingly, Western blot protein analysis of whole BMDM cell lysates treated with Aquamin and LPS did not result in a reduction in IκBα degradation (**Figures 3a, b**) or phosphorylation of p-65 subunit of the NF-κB complex (**Figures 3c, d**) compared to LPS alone. In this setting, early NF-κB activation events were assessed within minutes of LPS stimulation. Under these experimental conditions, Aquamin did not measurably alter IκBα degradation or p65 phosphorylation, suggesting that its suppressive effects on TNF-α, IL-6, and iNOS are unlikely to be mediated through the early NF-κB activation events examined downstream of LPS/TLR4 signaling. Early TLR4-mediated NF-κB activation is dependent on the recruitment of the adaptor protein MyD88, whereas sustained delayed NF-κB activation and IRF3 signaling require TLR4 endosomal trafficking and recruitment of the adaptor protein TRIF. We therefore investigated whether Aquamin altered the expression of the adaptor molecules *Myd88* and *Trif* also known as *TIcam1*. Stimulation of BMDMs with LPS resulted in increased expression of both *MyD88* and *Ticam1.* However, treatment with Aquamin specifically inhibited LPS-mediated *Ticam1* induction (**Figure 3e**) without affecting LPS-mediated *MyD88* gene expression levels (**Figure 3f**). These findings are consistent with Aquamin modulating TRIF-associated signaling downstream of TLR4 rather than altering the early NF-κB signaling events assessed in this study. However, because NF-κB activation also involves nuclear translocation, transcriptional activity, cofactor recruitment, and temporal regulation, additional time-course studies together with analyses of NF-κB nuclear localization and transcriptional activity will be required to fully determine whether Aquamin influences MyD88/NF-κB signaling.

### 3.4 Aquamin inhibits TRIF-dependent TLR3 signaling in murine BMDM

**Figure 4:**
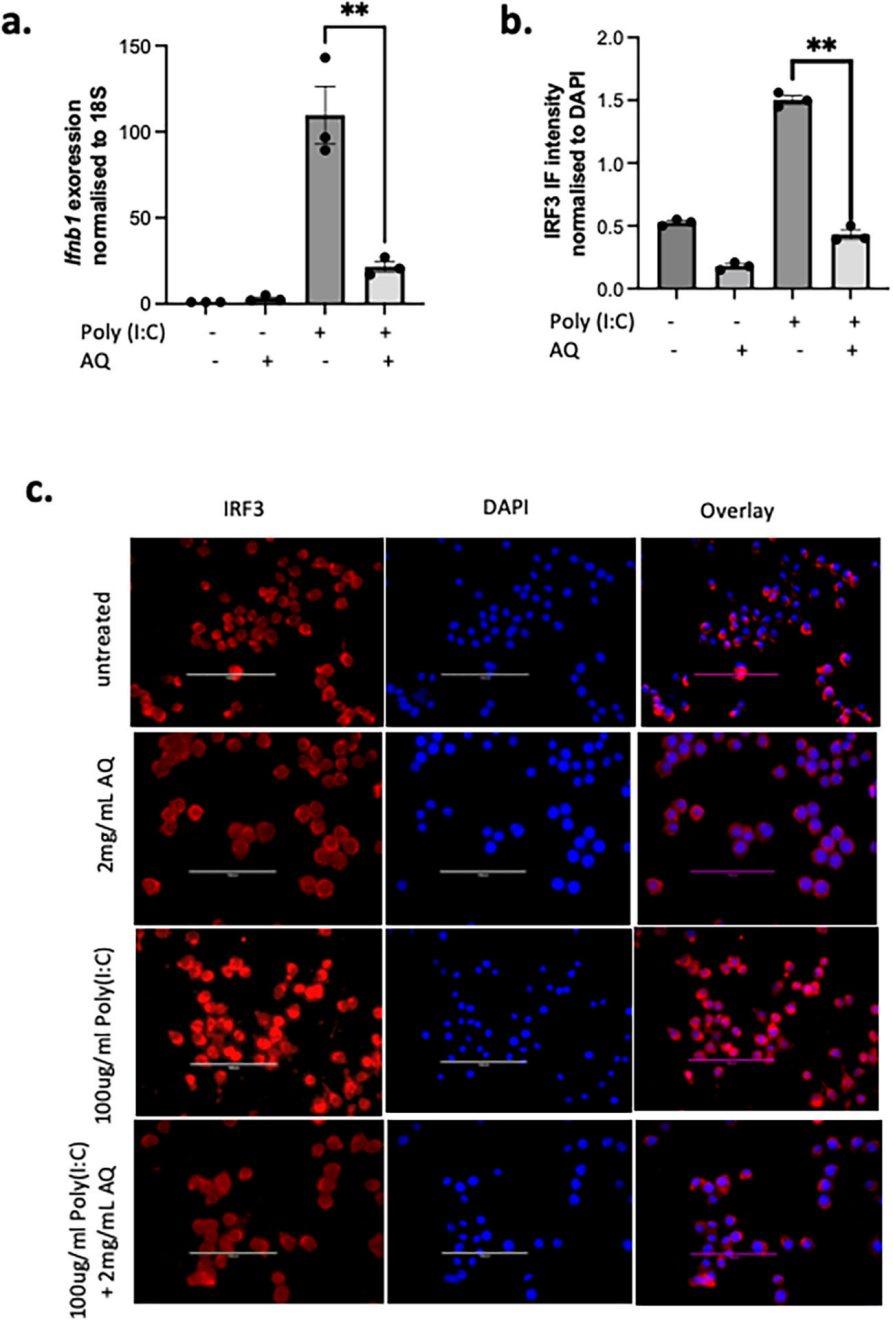
Aquamin reduced *Infb1* expression and IRF3 nuclear localization in Poly(I:C)-stimulated BMDM. BMDMs were pretreated with Aquamin (2 mg/mL) for 3 h before stimulation with Poly(I:C) (100 μg/mL) for 6 h. **(a)** *Ifnb1* gene expression normalized to 18S RNA, expressed relative to untreated cells. **(c)** Representative immunofluorescence (IF) of IRF3 protein abundance (red), DAPI nuclear stain (blue) and IF overlay in mBMDMs; scale bar, 100 μm. **(b)** Bar graph of IRF3 IF intensity normalized to DAPI. Data presented as mean ± SEM from three independent experiments. **p < 0.01 vs. Poly(I:C) alone using one-way ANOVA statistical test.

In order to further investigate the mechanism through which Aquamin inhibits innate TLR-driven responses, we next investigated whether Aquamin treatment elicited any effects on other TLR family members. Unlike TLR4, which stimulates both MYD88- and TRIF-dependent signaling pathways, TLR3 signaling in macrophages primarily uses the adaptor protein TRIF, without engaging MyD88 (8). To determine if Aquamin regulates TRIF-dependent IRF-3 signaling, investigations using Poly(I:C) stimulation were carried out to determine if Aquamin altered the mRNA expression of the IRF-3-dependent gene, *Ifnb1*. Interestingly, Aquamin reduced expression of *Ifnb1* at the mRNA level, post Poly(I:C) stimulation, when compared to Poly(I:C) treatment alone (**Figure 4a**). *Ifnb1* expression is dependent on activation of IRF-3 through phosphorylation events, followed by IRF3 dimerization and translocation to the nucleus, in macrophages after TLR3 stimulation. Therefore, we next analyzed the cellular localization of IRF3 expression in BMDMs under Poly(I:C) stimulation in the presence and absence of Aquamin (2 mg/mL) through immunofluorescent (IF) staining. As expected, Poly(I:C) increased the nuclear localization of IRF3 as evident by nuclear DAPI counterstaining compared to untreated and Aquamin only treated cells (**Figure 4**). A reduction in the intensity of IRF3 IF staining, as well as a reduction in the nuclear localization of IRF3, in Aquamin (2 mg/mL) pretreated cells was evident compared to cells treated with Poly(I:C) alone (**Figures 4b, c**). These results demonstrate that Aquamin can suppress canonical TLR3-dependent signaling responses, further supporting the idea that Aquamin can potentially modulate TLR signaling, downstream of the TRIF adaptor protein.

### 3.5 Aquamin inhibits TLR4 and TLR3 but not TLR9 cytokine release in murine BMDM

**Figure 5:**
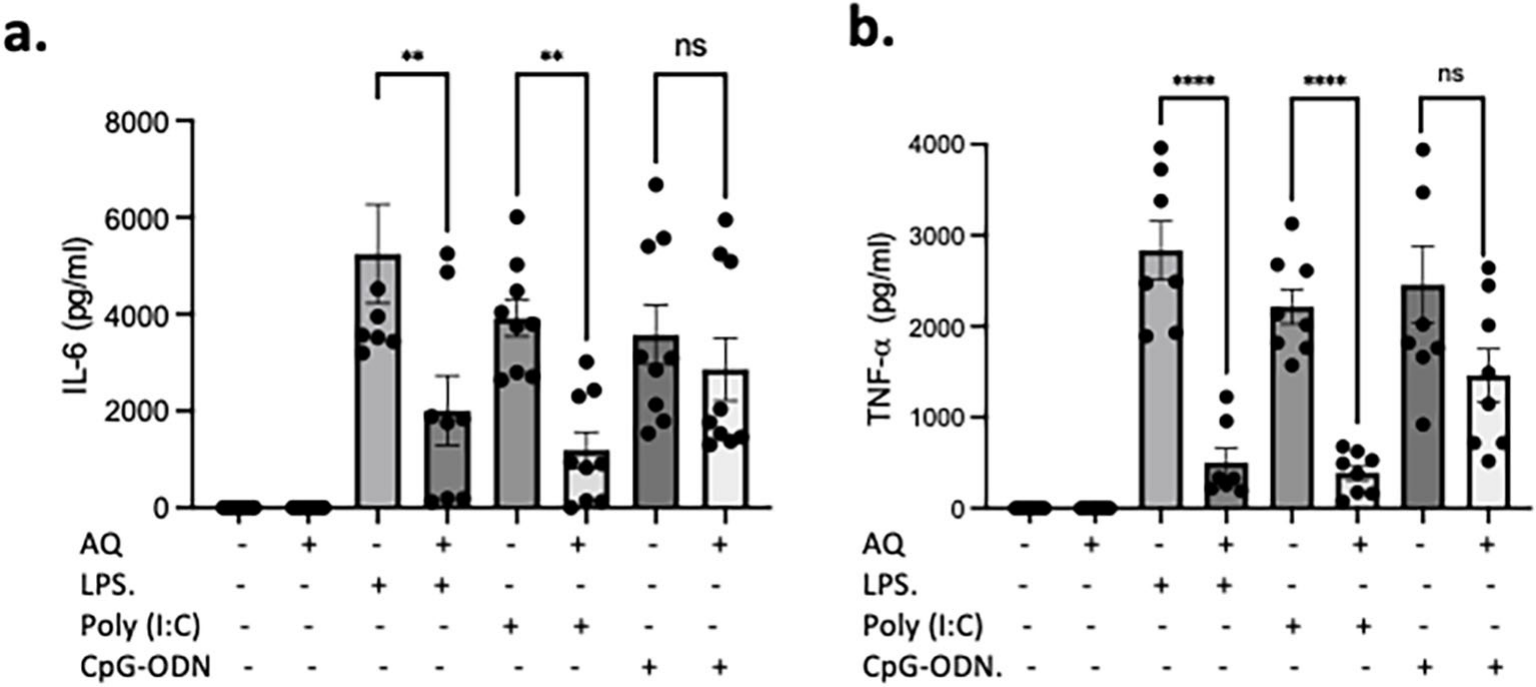
Aquamin inhibits TLR4- and TLR3-stimulated release of IL-6 and TNF-α in murine BMDMs without affecting TLR9. BMDMs pretreated with Aquamin (2 mg/mL) for 3 h prior to stimulation with the corresponding TLR agonists overnight, LPS (100 ng/mL) for TLR4 stimulation, Poly(I:C) (100 μg/mL) for TLR3 stimulation, and CpG (3 μM) for TLR9 stimulation. **(a)** IL-6 and **(b)** TNF-α cytokine levels in the cell culture supernatants measured by ELISA. Statistical comparisons were performed within each TLR agonist treatment group using one-way ANOVA followed by Dunnett’s multiple comparisons test, comparing Aquamin-treated cells with the corresponding agonist-alone control [LPS, Poly(I:C), or CpG, as appropriate]. *p*-values are indicated as **p < 0.01 and ****p < 0.0001. Data are expressed as mean ± SEM from three independent experiments.

Collectively, the data above demonstrate that Aquamin reduces cytokine release in human and murine innate cells under LPS and Poly(I:C) stimulation. These effects appear to occur in the absence of any detected inhibition of MyD88 expression, or activation of early downstream NF-κB-mediated signaling. While TLR3 primarily signals using the adaptor protein TRIF (8), TLR9, in contrast, signals exclusively using the adaptor protein MyD88 (9). Therefore, to gain a deeper understanding of Aquamin-mediated inhibition of TLR family signaling adaptor pathways, we examined whether Aquamin altered BMDM responses upon stimulation with specific agonists to TLR9 alongside TLRs 3 and 4. Primary murine BMDMs were pretreated with Aquamin for 3 h and exposed to specific TLR agonists, LPS for TLR4, Poly(I:C) for TLR3, and CpG DNA for TLR9 stimulation (**Figure 5**) for 12 h. As expected, all TLR agonists increased both IL-6 and TNF-α cytokine release from BMDMs compared to untreated cells after 12 h of stimulation. While Aquamin treatment significantly reduced IL-6 and TNF-α cytokine release from LPS and Poly(I:C) stimulation, the same inhibitory effect was not evident with Aquamin upon CpG DNA stimulation (**Figures 5a, b**). These data suggest that Aquamin is acting to modulate TRIF-dependent downstream signaling in response to TLR4 and TLR3 stimulation, without significantly impacting early MyD88-dependent signaling pathways downstream of TLR4 and TLR9.

## 4 Discussion

This study demonstrates that Aquamin exerts a consistent anti-inflammatory effect in primary innate immune cells by suppressing pro-inflammatory outputs downstream of TLR activation, with findings consistent with modulation of TRIF-associated signaling as a contributing mechanism. In both human PBMCs and murine BMDMs, Aquamin reduced LPS-induced secretion of key inflammatory cytokines TNF-α and IL-6 (**Figures 1a–d**), and in BMDMs, it also suppressed LPS-driven *Cxcl2* and *Ccl2* chemokine expression (**Figures 1e, f**). Importantly, these effects were not attributable to cytotoxicity (**Supplementary Figure 1**), indicating that Aquamin modulates inflammatory signaling rather than broadly impairing cell viability or function. Together, these findings support Aquamin as an immunomodulatory multi-mineral intervention, capable of dampening macrophage-driven inflammatory programs relevant to chronic inflammatory disease states. However, as Aquamin is a complex multi-mineral preparation, the relative contribution of its individual mineral components cannot be determined from the present study. Investigations incorporating mineral-matched controls, and physicochemical assessments of the culture conditions, including osmolarity and pH, will be important to distinguish mineral-specific effects from those attributable to the complete Aquamin formulation to further define the mechanisms underlying its anti-inflammatory activity.

A notable mechanistic insight from this work is that Aquamin’s suppression of LPS-induced cytokines and iNOS (**Figure 2**) appears uncoupled from inhibition of early canonical NF-κB activation events (**Figures 3a–d**). LPS stimulation classically activates the MyD88-dependent pathway rapidly, causing IκBα degradation and phosphorylation of NF-κB p65, which drives early transcription of inflammatory mediators (10). Here, Aquamin did not reduce IκBα degradation or p65 phosphorylation at early timepoints following LPS stimulation (**Figures 3a–d**), yet downstream inflammatory outputs (TNF-α, IL-6, and iNOS) were clearly reduced at later endpoints (**Figures 1**, **2**). This suggests Aquamin may not block proximal receptor engagement or the immediate MyD88→NF-κB activation cascade. Instead, the data are more compatible with Aquamin acting on later-phase transcriptional amplification, pathway crosstalk, or adaptor regulation mechanisms that can reduce cytokine accumulation even when early NF-κB activation is preserved (39).

Analysis of adaptor protein gene expression levels is consistent with Aquamin modulation of downstream signaling events that regulate later inflammatory responses. LPS increased both *Myd88* and *TRIF* gene expression in BMDMs, where Aquamin in the presence of LPS is shown here to selectively reduce *TRIF* induction without affecting *MyD88* expression (**Figures 3e, f**). The selective Aquamin-induced reduction in *TRIF* expression may provide a mechanistic basis for the observed immunosuppressive effects observed (**Figure 1**). These data may indicate that Aquamin dampens the endosomal/TRIF-dependent component of TLR4 signaling, which contributes to sustained NF-κB/MAPK activity and to IRF3-driven transcriptional gene regulation (11). The ability of Aquamin to modulate TRIF-dependent responses is further supported by Aquamin’s inhibitory effects observed under conditions of TLR3 stimulation, as TLR3 signals primarily through TRIF (12). Similar to LPS, Aquamin reduced Poly(I:C)-induced *Ifnb1* expression and decreased IRF3 transcription nuclear localization, indicating suppression of TRIF–IRF3 signaling pathways (**Figure 4**). Collectively, reduced *TRIF* expression, reduced IRF3 nuclear localization, and the reduced *Ifnb1* induction support the observation that Aquamin can modulate TRIF-associated signaling downstream of TLR3 and TLR4. However, these findings do not demonstrate direct inhibition of TRIF itself. Rather, they suggest that Aquamin may influence signaling events associated with TRIF, although additional mechanisms cannot be excluded. A deeper analysis will be required to determine whether Aquamin modulates defined TLR-dependent responses, including TRIF-associated signaling events such as TRIF protein abundance, TBK1/IKKϵ activation, and IRF3 phosphorylation or dimerization.

Notwithstanding this requirement, TLR pathway signaling selectivity was further indicated by the agonist cytokine comparison across TLR4, TLR3, and TLR9 stimulation. Aquamin inhibited cytokine release induced by LPS (TLR4) and Poly(I:C) (TLR3), but did not significantly suppress cytokine release induced by CpG (TLR9), which signals primarily through MyD88 (**Figure 5**). This pattern is consistent with Aquamin targeting distinct downstream mechanisms, including TRIF-dependent signaling, rather than globally suppressing TLR activation. Functionally, this is an important distinction as broad inhibition of TLRs can compromise antimicrobial defense and immune competence (40), whereas a more selective inhibitory approach may provide therapeutic benefit with a potentially more favorable immunological risk profile. While this study does not directly measure host defense outcomes, preservation of early NF-κB activation events and lack of effect on CpG-driven cytokines argue against nonspecific immunosuppression with Aquamin. Nevertheless, the present findings should be interpreted cautiously, as the molecular target responsible for these selective effects remains to be established.

Aquamin also markedly reduced LPS-induced iNOS protein expression in BMDMs (**Figure 2**). iNOS induction is a hallmark of pro-inflammatory macrophage activation and contributes to oxidative and nitrosative stress in tissues (37). Suppression of iNOS is therefore consistent with a shift away from an inflammatory macrophage phenotype and could be highly relevant in chronic inflammatory settings where sustained NO and RNS production exacerbates cellular damage by modifying proteins (S-nitrosylation) leading to potential barrier dysfunction and tissue injury in the gut (41). Given Aquamin’s reported benefits in gut (31, 32) and liver disease models (33), reduced iNOS (**Figure 2**) and chemokine expression (*Cxcl2* and *Ccl2*) (**Figures 1e, f**) may help explain how Aquamin decreases immune cell recruitment and inflammatory amplification within these tissue sites, supporting gut barrier integrity and limiting chronic damage. While the present study did not directly assess macrophage phenotype, future studies examining macrophage polarization markers and functional assays will be required to determine whether Aquamin promotes macrophage polarization toward a less inflammatory phenotype.

Although the findings demonstrate consistent modulatory effects of Aquamin in TLR signaling across species, the precise molecular target of Aquamin remains unresolved (29). Aquamin is a complex organo-mineral mixture with unique structures containing calcium, magnesium, and numerous trace elements, any of which could influence inflammatory signaling (27, 28). These may occur directly, for example, through kinase activity, phosphatase balance, or redox status (42), or indirectly, through membrane dynamics and receptor trafficking. One plausible mechanism, consistent with this data, is that Aquamin influences TLR4 endosomal trafficking or the formation and stability of TRIF-associated signaling complexes. Alternatively, Aquamin may regulate transcriptional control of *TRIF* itself or alter post-translational events required for IRF3 activation and nuclear translocation. Distinguishing among these possibilities will require targeted follow-up experiments assessing TRIF protein levels, IRF3 phosphorylation/dimerization, endosomal TLR4 localization, and measuring downstream signaling intermediates (e.g., TBK1/IKKϵ activation).

While this study provides novel insights into the capacity of Aquamin to suppress innate immune activation, the mechanistic conclusions are based primarily on adaptor mRNA regulation and functional readouts. A more comprehensive targeted assessment of TRIF protein abundance and activity would strengthen these conclusions. Furthermore, although early NF-κB signaling was evaluated using IκBα degradation and p65 phosphorylation, NF-κB activation also depends on nuclear translocation, transcriptional activity, co-factor recruitment, and MAPK-driven transcriptional synergy (39), which were not assessed in the present study. Similarly, while the findings are consistent with the modulation of TRIF-associated signaling, they do not establish whether Aquamin directly affects TRIF protein expression, TRIF signaling complex formation, TLR4 endocytosis, receptor trafficking, or downstream kinase activation. Future studies employing TRIF-deficient cells, selective pathway inhibitors, TBK1/IKKϵ inhibition, and assays of TLR4 endocytosis, together with assessment of IRF3 phosphorylation and dimerization, will be valuable for confirming pathway specificity and defining the molecular mechanism of Aquamin action. Furthermore, cell-type-specific profiling, including macrophage polarization assays, would clarify which immune cell populations are most responsive and whether Aquamin promotes broader anti-inflammatory programming (38).

**Figure 6:**
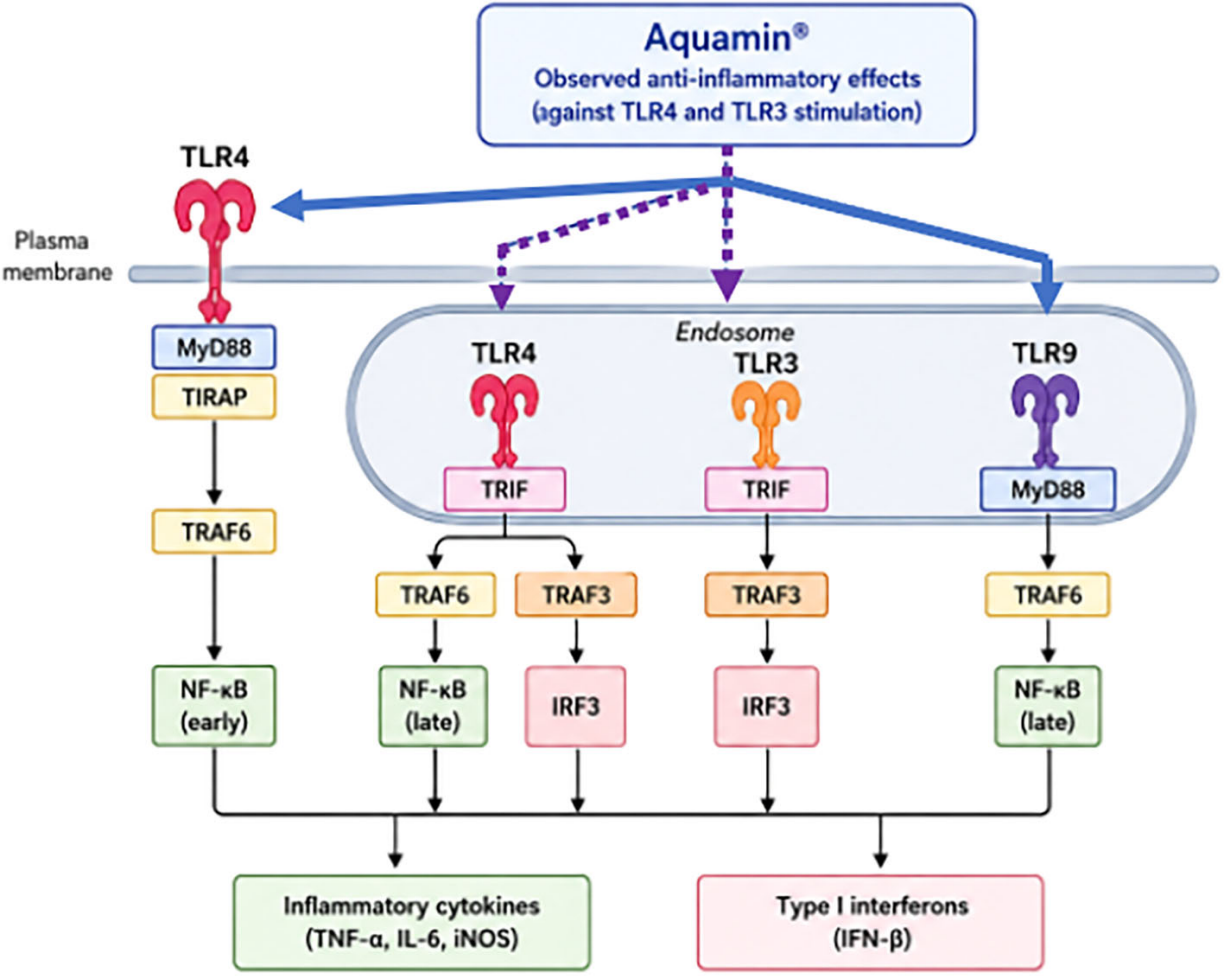
Simplified schematic of TLR4, TLR3, and TLR9 signaling pathways. Aquamin reduced pro-inflammatory cytokine release, *Ticam1 (Trif)* mRNA expression, IRF3 nuclear localization, and I*nfb1* gene expression downstream of TLR4 and TLR3 as indicated by purple dashed lines. No measurable effect on TLR4 *MyD88* gene expression, TLR4 early NF-kB activity, or TLR9-mediated cytokine responses as indicated by the blue solid line. These findings are consistent with the modulation of TRIF-associated TLR signaling; however, direct targeting of TRIF or its signaling complex was not demonstrated.

In conclusion, Aquamin robustly suppresses TLR-driven inflammatory mediator production in primary human and murine innate immune cells (**Figure 6**) and provide a mechanistic framework that aligns with Aquamin’s reported protective effects in inflammatory disease contexts (31, 32). These findings provide a rationale for further mechanistic investigations of Aquamin to define the proximal molecular target(s), at the protein and signaling-complex level, and validating these effects in relevant *in vivo* models. Although the concentrations of Aquamin used in this study demonstrated clear biological activity *in vitro*, their relationship to systemic concentrations achieved following oral supplementation remains unknown. Consequently, caution should be exercised when extrapolating these findings to the *in vivo* setting. With Aquamin administration orally, substantially higher local concentrations may be achieved within the gastrointestinal lumen. As such, the concentrations used in the present study may be particularly relevant to investigating mechanisms underlying Aquamin’s reported beneficial effects on intestinal health (32). However, future pharmacokinetic, tissue distribution, and dose–response studies will be required to establish the broader *in vivo* relevance and translational significance of the concentrations used in this study. These investigations will be essential to establish Aquamin’s therapeutic potential as a nutraceutical strategy for controlling pathological inflammation while preserving core innate immune responsiveness.

## Data Availability

The original contributions presented in the study are included in the article/**Supplementary Material**. Further inquiries can be directed to the corresponding author.
