# Supplementary material for: Aquamin, a marine derived multi-mineral, attenuates toll-like receptor-mediated inflammatory responses in macrophages
Source: Front Immunol. 2026 Aug 4;17:1901355. doi: 10.3389/fimmu.2026.1901355 (PMC13481773; doi:10.3389/fimmu.2026.1901355)
Supplement: Supplementary file 1 [file DataSheet1.pdf]

Supplemental Table 1. Mineral composition of Aquamin®

Element	Amount	Element	Amount	Element	Amount
Carbon	255,000 ppm	Indium	<0.001 ppm	Samarium	0.035 ppm
Aluminum	22.6 ppm	†Iodine	0.41 ppm	Scandium	1.209 ppm
Antimony	<0.5 ppm	Iridium	0.002 ppm	Selenium	<0.5 ppm
Arsenic	0.101 ppm	Iron	80.1 ppm	Silicon	9.44 ppm
Barium	4.06 ppm	Lanthanum	0.899 ppm	Silver	1.28 ppm
Beryllium	1.60 ppm	Lead	0.019 ppm	Sodium	2,055 ppm
Bismuth	<0.5 ppm	Lithium	<0.5 ppm	Strontium	968 ppm
Boron	13.8 ppm	Lutetium	0.012 ppm	Sulfur	1,144 ppm
Cadmium	0.268 ppm	Magnesium	1.05%	Tantalum	0.015 ppm
Calcium	12.10%	Manganese	17.03 ppm	Tellurium	6.67 ppm
Cerium	0.225 ppm	Mercury	0.004 ppm	Terbium	0.008 ppm
Cesium	<0.001 ppm	Molybdenum	0.773 ppm	Thallium	<0.5 ppm
†Chloride	1,852 ppm	Neodymium	0.143 ppm	Thorium	3.09 ppm
Chromium	1.27 ppm	Nickel	0.605 ppm	Thulium	0.005 ppm
Cobalt	1.51 ppm	Niobium	7.77 ppm	Tin	0.215 ppm
Copper	2.01 ppm	Osmium	<0.001 ppm	Titanium	2.72 ppm
Dysprosium	0.046 ppm	Palladium	0.143 ppm	Tungsten	<0.5 ppm
Erbium	0.033 ppm	Phosphorus	20.4 ppm	Vanadium	<0.5 ppm
Europium	0.012 ppm	Platinum	0.008 ppm	Ytterbium	0.030 ppm
†Fluoride	2.099 ppm	Potassium	83.2 ppm	Yttrium	2.71 ppm
Gadolinium	0.039 ppm	Praseodymium	0.031 ppm	Zinc	1.76 ppm
Gallium	0.037 ppm	Rhenium	0.001 ppm	Zirconium	3.48 ppm
Germanium	<0.001 ppm	Rhodium	0.147 ppm		
Gold	<0.5 ppm	Rubidium	0.011 ppm		
Hafnium	0.074 ppm	Ruthenium	0.473 ppm		
Holmium	0.010 ppm				

The trace mineral composition of Aquamin® TG was determined by an independent laboratory (Advanced Laboratories, Inc., Salt Lake City) for Marigot Limited (Ireland) in 2018.

Individual trace element composition was determined by Inductively Coupled Plasma Optical Emission Spectroscopy (ICPOES) except for carbon (determined by ASTM D-1552), chloride and iodine (determined by Titration), and fluoride (determined by AOAC 939.11).
