# Supplementary material for: Aquamin, a marine derived multi-mineral, attenuates toll-like receptor-mediated inflammatory responses in macrophages
Source: Front Immunol. 2026 Aug 4;17:1901355. doi: 10.3389/fimmu.2026.1901355 (PMC13481773; doi:10.3389/fimmu.2026.1901355)
Supplement: Supplementary file 2 [file DataSheet2.pdf]

Supplemental Table 2: Microbiological testing screen for Aquamin

Total viable count	5,000cfu/g max.
Yeast & Moulds	100cfu/g max.
E. Coli	Absent in 1g
Coliforms	Absent in 1g
Enterobacteriaceae	Absent in 1g
Staphylococcus aureus	Absent in 1g
Salmonella	Absent in 25g
