# Supplementary material for: Aquamin, a marine derived multi-mineral, attenuates toll-like receptor-mediated inflammatory responses in macrophages
Source: Front Immunol. 2026 Aug 4;17:1901355. doi: 10.3389/fimmu.2026.1901355 (PMC13481773; doi:10.3389/fimmu.2026.1901355)
Supplement: Supplementary file 3 [file DataSheet3.pdf]

Supplemental Table 3 – SYBR® Green primer sequence

Gene Name		Gene Sequence
GAPDH	Forward	5'- AAC AGC AAC TCC CAC TCT TC- 3'
	Reverse	5'- CCT GTT GCT GTA GCC GTA TT- 3'
TNF-alpha	Forward	5'-CTA CCT TGT TGC CTC CTC TTT-3'
	Reverse	5'- GAG CAG AGG TTC AGT GAT GTA G-3'
IL-6	Forward	5'- GTC TGT AGC TCA TTC TGC TCT G-3'
	Reverse	5'- GAA GGC AAC TGG ATG GAA GT-3'
TRIF	Forward	5'- GGA CCT CAG CCT CTC ATT ATT C-3'
	Reverse	5'-AGG TTC CCT TCC TCC ACT AT-3'
MyD88	Forward	5'- GTA TCC TGC GGT TCA TCA CTA T-3"
	Reverse	5'- GAA CTC TTC CAC TCA GCT ATC C-3'
CXCL2	Forward	5'-CCA TTG CCC AGA TGT TGT TAT G -3'
	Reverse	5' GCC ATC CGA CTG CAT CTA TT-3'
CCL2	Forward	5'- GAA GGA ATG GGT CCA GAC ATA C -3'
	Reverse	5'- TCA CAC TGG TCA CTC CTA CA -3'
IFN-beta	Forward	5'-GGA AAG ATT GAC GTG GGA GAT-3'
	Reverse	5'-CAG GCG TAG CTG TTG TAC TT-3'
18S RNA	Forward	5'-CTG AGA AAC GGC TAC CAC ATC-3'
	Reverse	5'-GCC TCG AAA GAG TCC TGT ATT G-3'
